# Faecal Immunochemical Testing to Detect Colorectal Cancer in Symptomatic Patients: A Diagnostic Accuracy Study

**DOI:** 10.3390/diagnostics13142332

**Published:** 2023-07-10

**Authors:** Farzana Rahman, Mihir Trivedy, Christopher Rao, Funmi Akinlade, Ahmer Mansuri, Atul Aggarwal, Faidon-Marios Laskaratos, Nirooshun Rajendran, Saswata Banerjee

**Affiliations:** 1 Barking, Havering and Redbridge University Hospitals NHS Trust, Romford RM7 0A, UK; farzana.rahman12@nhs.net (F.R.);; 2 Barts Health NHS Trust, Whipps Cross Hospital, London E11 1NR, UK; 3 Department of Surgery and Cancer, Imperial College London, London SW7 2BX, UK; 4 North Cumbria Integrated Care NHS Foundation Trust, Carlisle CA2 7HY, UK

**Keywords:** faecal immunochemical testing, colorectal cancer, diagnostic accuracy study, primary healthcare

## Abstract

(1) Background: NHS England recommended faecal immunochemical testing (FIT) for symptomatic patients in June 2020 to rationalise limited diagnostic services during COVID-19. (2) Aim: to investigate the diagnostic performance of FIT, analysing the proportion of FIT-negative colorectal cancers (CRC) missed in symptomatic patients and how this risk could be mitigated. (3) Design and Setting: a retrospective study of biochemistry and cancer databases involving patients referred from primary healthcare with suspected CRC to a single secondary care trust in North East London. (4) Methods: a retrospective cohort diagnostic accuracy study was undertaken to determine the performance of FIT for detecting CRC at 10 µgHb/g. (5) Results: between January and December 2020, 7653 patients provided a stool sample for FIT analysis; 1679 (22%) samples were excluded due to inadequate or incorrect specimens; 48% of suspected CRC referrals completed FIT before evaluation; 86 FIT tested patients were diagnosed with histologically proven CRC. At 10 µgHb/g, FIT performance was comparable with the existing literature with a sensitivity of 0.8140 (95% CI 0.7189–0.8821), a specificity of 0.7704 (95% CI 0.7595–0.7809), a positive predictive value (PPV) of 0.04923 (95% CI 0.03915–0.06174), a negative predictive value (NPV) of 0.9965 (95% CI 0.9943–0.9978), and a likelihood ratio (LR) of 3.545; 16 patients with CRC had an FIT of ≤10 µgHb/g (18.6% 95% CI 11.0–28.4%). (6) Conclusions: this study raises concerns about compliance with FIT testing and the incidence of FIT-negative CRC at the NICE recommended threshold and how this risk can be mitigated without colonic imaging. Whilst FIT may have facilitated prioritisation during COVID-19, we must be cautious about using FIT alone to determine which patients are referred to secondary care or receive further investigation.

## 1. Introduction

The 2019 novel coronavirus disease (COVID-19) pandemic has compounded existing challenges faced by UK endoscopy services. Prior to the pandemic, referrals for CRC almost doubled over a 5-year period [1], and over 40% of endoscopy units were failing to comply with colorectal cancer (CRC) national diagnostic standards [2]. At the peak of the first wave of the COVID-19 pandemic, there was a 92% reduction in the volume of colonoscopies performed [3]. It was estimated that it would have been necessary to increase UK endoscopy capacity to 130% to eliminate the backlog of cases by June 2022 [4].

In 2017, the National Institute of Health and Care Excellence (NICE) recommended faecal immunochemical testing (FIT) for the detection of CRC in patients with low-risk symptoms [5] (Table 1). There has subsequently been considerable interest in using it as a tool to stratify the risk of CRC in patients with higher-risk symptoms in the context of limited existing diagnostic capacity and the increasing volume of referrals with suspected CRC [1,6,7,8,9]. In patients with high-risk symptoms for CRC, FIT performs well, with several large trials suggesting that FIT has an 87–94% sensitivity, 80–89% specificity, 12–18% positive predictive value (PPV), and 99% negative predictive value (NPV) at a threshold of 10 µgHb/g [1,6,7,9,10]. The diagnostic backlog resulting from the COVID-19 pandemic has increased the importance and relevance of this work [11]. In June 2020, NHS England published guidance to mitigate the risk of missing CRC diagnosis during the first wave of the pandemic, recommending that all patients with suspected CRC should have an FIT test and that those with an FIT < 10 µgHb/g did not need urgent investigation [12].

The Barking, Havering, and Redbridge University Hospitals NHS Trust manages two acute general hospitals in outer North East London: Queen’s Hospital and King George Hospital. Collectively, they serve a population of more than 750,000 people, approximately 8% of London’s population, including some of the most economically deprived populations in England and communities where English language proficiency is lower than the national average [13,14,15]. Primary care faces significant challenges within the clinical commissioning groups (CCG) served by our trust, with an average list size approximately 25% greater than the national average [16]. We sought to investigate whether the diagnostic performance of FIT in our population following implementation of the June 2020 NHS England guidance [12] was comparable to performance in large clinical studies [1,8,17,18]. We hoped this would inform what role FIT testing should have in the diagnostic pathway for patients with suspected CRC during and following recovery from the COVID-19 pandemic.

## 2. Materials and Methods

### 2.1. Patient Population, Inclusion and Exclusion Criteria

From May 2019 onwards, all adult patients referred to secondary care with suspected CRC with low-risk symptoms defined by NICE DG30 criteria [5] were asked to complete a FIT test. From June 2020 onwards, all patients, including those with higher-risk symptoms defined by NICE NG12 criteria [19], were asked to provide an FIT sample to their primary care provider. This was in order to facilitate risk stratification and prioritisation of CRC diagnostic testing during the peak of the COVID-19 pandemic. 

**Table 1:** High-risk symptoms of colorectal cancer (as defined by NICE NG12 criteria [19]) vs. low-risk symptoms (as defined by NICE DG30 criteria [5]).

High-Risk Symptoms (NG12)	Low-Risk Symptoms (NG12, DG30)
Unexplained anal mass or ulceration	Patient aged ≥50 with unexplained abdominal pain
Unexplained rectal or abdominal mass	Patient aged ≥50 with unexplained weight loss
Patient aged ≥40 with unexplained abdominal pain and weight loss	Patient aged <60 with an unexplained change in bowel habits
Patient aged ≥50 with unexplained rectal bleeding	Patient aged <60 with unexplained iron-deficiency anaemia
Patient aged ≥60 with iron-deficiency anaemia	Patient aged ≥60 with anaemia of any aetiology
Patient aged ≥60 with an unexplained change in bowel habits	
Patient aged ≥60 with unexplained blood in the stool	

All patients referred on the basis of a suspected lower-gastrointestinal cancer pathway (two-week wait, 2WW) who completed an adequate FIT test between January 2020 and December 2020 were included in this analysis. It is noted that the guidance for FIT testing changed during the time period covered by this study; in June 2020, all patients, regardless of whether they were low-risk or high-risk, were asked to provide an FIT test, while previously this was required only for those with low-risk symptomatology. Despite this, all patients in our dataset had an FIT test performed prior to further imaging and are therefore included in the analysis. Patients who presented as an emergency who had previously supplied an FIT sample were also included. Patients who did not provide a labelled sample, completed an inadequate sample, or for whom it was not possible to analyse the sample for any other reason were excluded from the analysis. Only patients with a histologically proven diagnosis of adenocarcinoma of the colon or rectum were considered to have CRC. Demographic characteristics and clinical information were obtained from electronic patient records that were populated as part of routine patient care.

### 2.2. Bichemical Sample Analaysis

FIT samples were analysed at King George Hospital, Goodmayes using the Eiken OC-Sensor FIT-Screening System (Mast Group Ltd., Bootle, UK) with a measuring range from 6 µgHb/g to 200 µgHb/g. A positive FIT was defined as greater than or equal to the NICE recommended threshold level of 10 µgHb/g [5]. Patients with an FIT greater than 100 µgHb/g on referral were identified as potentially having a higher risk of CRC. 

### 2.3. Statistical Analysis

Statistical analysis was undertaken using GraphPad Prism Version 9 for Windows (GraphPad Software, San Diego, CA USA) and Microsoft Excel for Microsoft 365 (Microsoft Corporation, Redmond, WA, USA). A contingency table was used to calculate sensitivity, specificity, negative predictive value (NPV), positive predictive value (PPV), and likelihood ratio (LR) at 10 µgHb/g and 100 µgHb/g. The Wilson–Brown method was used to calculate 95% CI [20]. The proportion of patients with CRC and a negative FIT test was also calculated, and 95% CI was estimated using the Clopper–Pearson exact method [21]. A receiver operator curve (ROC) was also constructed, and the area under the curve (AUC) was calculated with confidence intervals using the Wilson–Brown method [20]. The Youden index (J) was used to define the optimal cut point [22]. Fisher’s exact test was used to compare proportions [23].

## 3. Results

### 3.1. Included Patients

Between January 2020 and December 2020, 7653 patients provided a stool sample in primary care for FIT analysis; 1679 (22%) samples were excluded from analysis due to inadequate or incorrect specimens. Subsequently, of the remaining 5974 samples, 1422 (24%) samples were equal to or above the NICE mandated guidelines of 10 µgHb/g, constituting a positive FIT, while 4552 (76%) were an FIT value of ≤9 µgHb/g, constituting a negative FIT.

The new amended referral standards [12] had an impact on the proportion of patients completing FIT. Of the 3536 referrals through the urgent suspected colorectal cancer pathway after June 2020, only 1684 (48%) of patients completed an FIT before clinical assessment. 

### 3.2. Diagnostic Performance of FIT

**Figure 1:**
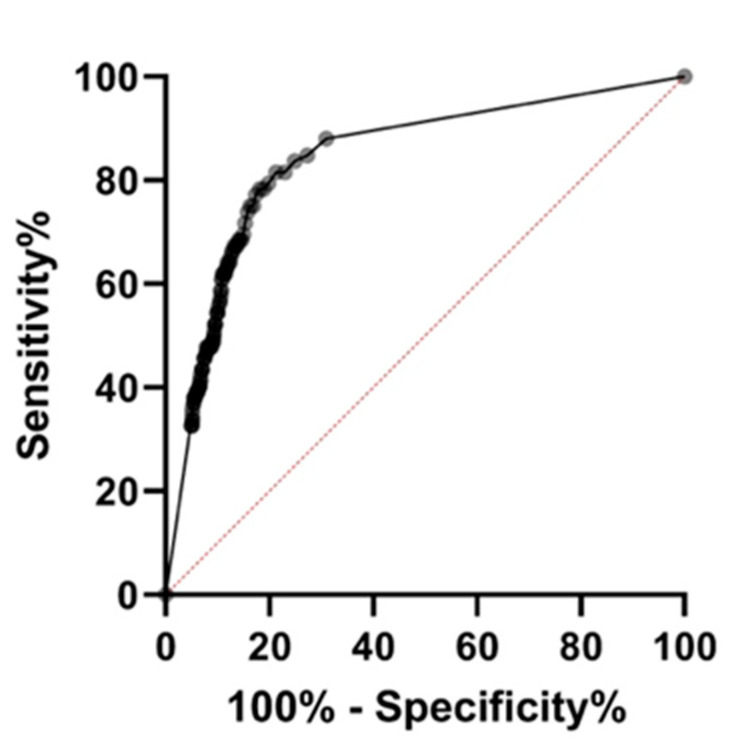
Summary receiver operator curve for the use of faecal immunochemical testing to detect colorectal cancer. By convention, the Y axis shows the % sensitivity defined as the % of patients who have the target disease (colorectal cancer) who will test positive for colorectal cancer at a given FIT threshold. The X axis shows 100%—the % of patients who do not have the target disease (colorectal cancer) who will test negative for colorectal cancer using FIT testing at the corresponding threshold. This demonstrates the trade-off between the sensitivity (the ability of FIT to detect all cases of colorectal cancer) and the specificity (the ability of FIT to differentiate patients who do and do not have colorectal cancer).

Exploration of local cancer databases found that 86 patients were later diagnosed with a histologically proven adenocarcinoma of colorectal origin, accounting for 1.4% of all patients who completed an FIT (Table 2). A ROC curve was constructed (Figure 1), and the AUC was 0.8422 (95% CI 0.7995–0.8849). The maximum value of J was 0.6019 corresponding to a FIT threshold of 11µgHb/g, and therefore sensitivity, specificity, NPV, PPV, and LR are reported for thresholds of 10 µgHb/g, 11 µgHb/g, and 100 µgHb/g (Table 2). At a threshold of 10 µgHb/g, the performance of FIT for the detection of CRC in our study was comparable with existing literature with a sensitivity of 0.8140 (95% CI 0.7189–0.8821), a specificity of 0.7704 (95% CI 0.7595–0.7809), a PPV of 0.04923 (95% CI 0.03915–0.06174), an NPV of 0.9965 (95% CI 0.9943–0.9978), and an LR of 3.545.

Of the patients with a positive FIT result, 347 (24%) were not referred to secondary care. The reasons for this are multiple, including patients deemed too unwell or declining to undergo invasive investigation.

### 3.3. Incidence of FIT-Negative CRC 

Of the 86 patients diagnosed with CRC, 16 (18.6% 95% CI 11.0–28.4%) had a negative FIT at the NICE recommended threshold of 10 µgHb/g (Table 2). Ten of the 16 FIT negative CRC had right-sided tumours and seven had anaemia on presentation (Table 3). The proportion of patients presenting with anaemia in the FIT-negative CRC group (7/16) (Table 4) was not significantly different from the FIT-positive CRC group (37/70) (*p* = 0.5857).

## 4. Discussion

This retrospective cohort diagnostic accuracy study evaluated the performance of FIT as an essential requirement in the urgent suspected CRC referral pathway during the COVID-19 pandemic within a North East London population. Amongst the 7653 patients that provided a stool sample to primary care, a large proportion of samples were not analysed due to inadequate specimens, requiring further samples to be sent, potentially leading to delays in diagnoses. There are many potential reasons for this, though it is appreciated that using FIT for the new purpose of stratifying imaging for CRC diagnosis will undoubtedly have contributed. However, amongst the 5974 that were analysed, CRC was later diagnosed in 86 patients (1.4%).

This study demonstrates that FIT has performed effectively at higher thresholds, with 10% of patients diagnosed with CRC at 100 µgHb/g (Table 2). At this level, FIT is highly specific for CRC (0.9290 95% CI 0.9222–0.9353) with an LR of 6.552, highlighting the utility of FIT at higher levels for the prioritisation of diagnostic studies both during the COVID-19 pandemic and potentially also during the recovery from the pandemic.

This differed from the performance of FIT at lower thresholds, where 16 of the 86 patients were considered FIT-negative at the NICE threshold of 10 µgHb/g. Therefore, approximately 20% of CRC may have been missed at the NICE threshold of 10 µgHb/g within our catchment population.

### 4.1. Comparison with the Published Literature

The significant number of stool samples excluded from the FIT analysis was not congruent with the published literature that reports that FIT is well accepted [1,9] and may reflect the challenging nature of our local population and healthcare infrastructure. It may also reflect the impact of the lockdown during the first wave of the COVID-19 pandemic. Nevertheless, further research is necessary to understand the reasons why patients did not find FIT acceptable or easy to comply with, exploring whether socio-linguistic factors may be responsible.

However, of those included, approximately 20% of patients with CRC diagnosed in our institution had negative FIT results at the NICE recommended threshold of 10 µgHb/g. This is higher than demonstrated by previous research that implies roughly 10% of CRC are FIT-negative at colonoscopy [1,10,23]. Both of these figures raise concerns about discharging patients or not referring individuals to secondary care based solely on FIT results. 

It has also been previously reported that most FIT-negative CRC patients had iron deficiency anaemia and right-sided cancers [24]. It has been suggested that the risk of missing an FIT-negative CRC can be mitigated by investigating patients presenting with anaemia. Disappointingly, this was not our experience, as a significant proportion of FIT-negative CRC patients did not present with anaemia or have right-sided cancers at our institution. 

It has been suggested that lowering the threshold for a positive test in FIT-positive patients to the limits of detection may improve the sensitivity of FIT [8]; in our study over 10% of patients with CRC would still have been missed. Furthermore, the effect of lowering the FIT threshold needs to be balanced against the effect that this would have on the demand for diagnostic studies, and therefore it has been suggested that individualised patient thresholds should be adopted [25]. Recent studies have suggested that repeat stool sampling may improve the diagnostic performance of FIT [26,27,28]. This strategy has been incorporated into recent NHS England guidelines [29], and work is currently underway to investigate the performance of FIT in these circumstances in our local population.

It has been suggested that FIT-negative patients with suspected CRC can be safety-netted within primary care, removing the need for urgent referral to specialist centres for diagnostic studies. In our study, the percentage of patients with CRC who were FIT-negative was 18.4%, suggesting the need for a very tight safety net. The merits of a strategy which places the burden of safety netting FIT-negative patients with suspected CRC on primary care services, without consideration of local circumstances, is questionable. In areas where primary care was under-resourced and challenged prior to the COVID-19 pandemic, the situation following the COVID-19 pandemic is critical [30,31,32]. Given the difficulties in mitigating against the risk of missing FIT-negative CRC and the absence of clear consensus in the literature about how this should be undertaken, it does not seem appropriate that primary care clinicians should be compelled to accept this responsibility, and we suggest that a collaborative approach between primary and secondary care is necessary to develop an integrated pathway for the safety netting of FIT-negative patients.

### 4.2. Strengths and Limitations of This Study

This diagnostic accuracy study has many strengths, including a large cohort of patients contributing to FIT analysis. Additionally, all stool samples were collected and analysed using a single analyser with an extensive measuring range (6 µgHb/g to 200 µgHb/g). This had many benefits, allowing this study to investigate the performance of FIT at different thresholds, even lowering the NICE threshold to 6 µgHb/g to study if this could improve its performance in detecting CRC. Furthermore, this study collected data from biochemistry databases, which incorporated results and initial presenting symptoms to general practitioners, allowing the authors to explore any correlations between presentations and FIT results.

However, this study had its limitations; disappointingly, only 48% of patients completed an FIT before clinical investigation and assessment in secondary care. In addition, a considerable proportion of patients sent a stool sample, however, the samples were not all processed when received at the biochemistry laboratory, for several reasons. These include inadequate labelling of specimens, patients not being provided with correct FIT tubes, and lack of patient understanding of how to offer FIT samples. This raises concerns about the practicalities, potential missed CRC, and probable delays in diagnosis and treatment in this group of patients due to the necessity to repeat the process. There is a clear lack of congruence between the proportion of unsuccessful FIT tests in our population versus the population at large (where FIT is generally well utilised). There are many potential reasons for this. The population served by our hospitals is one of the most economically deprived in the United Kingdom, with literacy rates below the national average, and furthermore, the average level of English language proficiency is again lower than the national average [13,14,15]. This can be ameliorated by use of diagram-heavy information leaflets or by presenting patients with a number of different language translations of the instruction leaflet.

Furthermore, this study suffers the limitations of a retrospective study. For example, a significant proportion of FIT-negative patients were not referred during the peak of the COVID-19 pandemic. Whilst this may have adversely affected the reported diagnostic performance of FIT, this should not have fundamentally changed the proportion of FIT-negative CRC and may in fact have improved it. The selective use of colonoscopy with a greater reliance on cross-sectional imaging during the first peak of the COVID-19 pandemic in FIT-positive patients (particularly high-risk individuals) may, however, have affected CRC detection rates and therefore adversely affected the proportion of FIT-negative CRC. It is therefore possible that concerns about the acceptability and safety of FIT testing raised by this dataset collected during the first wave of the COVID-19 pandemic may not be relevant as the NHS recovers from the pandemic. Finally, we were unable to report the performance of FIT in our institution for the diagnosis of other significant colorectal pathologies such as high-risk adenomas and inflammatory bowel disease.

## 5. Conclusions

This study represents a real-world assessment of the utility of FIT in the urgent referral pathway for symptomatic patients with suspected CRC. It highlights limitations of FIT testing such as poor patient compliance in some populations. It also raises concerns about the incidence of FIT-negative CRC at the NICE recommended threshold. Whilst FIT-negative CRCs were evident in previously reported large clinical studies, they appear to be a significantly greater problem in this real-world dataset. Therefore, whilst FIT may have performed well to risk-stratify patients when diagnostic resources were limited during the COVID-19 pandemic, its real-world utility in symptomatic patients must be interpreted cautiously, and further research is needed.

When the real-world performance of FIT is evaluated further, the authors would suggest population-specific strategies to improve compliance with FIT testing, an integrated strategy for safety netting FIT-negative patients developed by primary and secondary care services, the use of repeated FIT sampling for FIT-negative patients, and the investigation of all patients with iron-deficiency anaemia and symptoms consistent with sub-acute bowel obstruction.

## Figures and Tables

**Table 2:** Number of colorectal cancers detected according to faecal immunochemical level. CI = confidence intervals, CRC = colorectal cancer, FIT = faecal immunochemical testing, Hb = haemoglobin.

FIT Test Result	True Positive	False Positive	True Negative	False Negative	Fit Negative CRC
10 µgHb/g Threshold	70	1352	4536	16	18.6% (95% CI 11.0–28.4%)
11 µgHb/g Threshold	70	1256	4632	16	18.6% (95% CI 11.0–28.4%)
100 µgHb/g Threshold	40	418	5470	46	53.5% (95% CI 42.4–64.3%)

**Table 3:** Sensitivity, specificity, positive predictive value, negative predictive value, and likelihood ratio of faecal immunochemical testing for detection of colorectal cancer. CI = confidence interval, Hb = haemoglobin, PPV = positive predictive value, NPV = negative predictive value.

Threshold for Positive Test	10 µgHb/g in Either Test (95% CI)	11 µgHb/g in Both Tests (95% CI)	100 µgHb/g in Both Tests (95% CI)
Sensitivity	0.8140 (0.7189–0.8821)	0.8140 (0.7189–0.8821)	0.4651 (0.3635–0.5698)
Specificity	0.7704 (0.7595–0.7809)	0.7867 (0.7760–0.7970)	0.9290 (0.9222–0.9353)
PPV	0.04923 (0.03915–0.06174)	0.05279 (0.04199–0.06617)	0.08734 (0.06479–0.1167)
NPV	0.9965 (0.9943–0.9978)	0.9966 (0.9944–0.9979)	0.9917 (0.9889–0.9937)
Likelihood Ratio	3.545	3.816	6.552

**Table 4:** Clinical Characteristics of colorectal cancers detected with negative faecal immunochemical tests. CI = confidence intervals, CRC = colorectal cancer, FIT = faecal immunochemical testing, Hb = haemoglobin, MCV = mean cell volume.

Age Gender	FIT Result µg Hb/g	Presenting Symptoms	Diagnosis	Emergency Presentation	Anaemia	Hb g/L	MCV fL	Platelets 10^9^/L	Ferritin ug/L
85 Male	9	Anaemia	Adenocarcinoma of hepatic flexure T2N0M0	No	Yes	81	71.4	507	9
45 Female	8	Change in bowel habits, rectal bleeding	Adenocarcinoma of rectum T3N1M0	No	No	124	86.5	312	76
93 Male	7	Anaemia, change in bowel habits	Adenocarcinoma of ascending colon, T2N0M0	No	Yes	70	66.5	306	7
55 Male	7	Abdominal pain, anaemia, weight loss	Adenocarcinoma of transverse colon T4N2M1	No	Yes	123	80.7	239	27
54 Male	7	Abdominal pain, anaemia, change in bowel habits	Adenocarcinoma of caecum T4N2M1	Yes	Yes	127	80.2	410	261
87 Female	6	Change in bowel habits, abdominal pain	Adenocarcinoma of ascending colon, T3N2M1	No	No	120	79.3	590	
80 Female	6	Rectal bleeding	Adenocarcinoma of rectum, no surgery	No	No	131	89.5	310	73
67 Female	<6	Anaemia, change in bowel habits	Adenocarcinoma of caecum T4N2M0	Yes	Yes	99	97.5	198	160
46 Male	<6	Obstruction	Adenocarcinoma of transverse colon T2N2M0	Yes	No	146	87.8	268	134
79 Male	<6	Abdominal pain, anaemia	Adenocarcinoma of hepatic flexure T3N1M0	No	Yes	126	89.7	199	44
79 Female	<6	Change in bowel habits, weight loss	Adenocarcinoma of caecum T4N2M1	No	No	133	87.9	310	669
44 Female	<6	Anaemia	Adenocarcinoma of caecum T4N2M1	No	Yes	107	73.5	733	-
59 Male	<6	Change in bowel habits	Adenocarcinoma of splenic flexure T2N0M1	No	No	169	86.7	180	192
57 Female	<6	Change in bowel habits	Adenocarcinoma of caecum T1N0M0	No	No	134	90.9	312	103
56 Female	<6	Change in bowel habits	Adenocarcinoma of ascending colon T2N0M0	No	No	139	94.6	213	41
70 Female	<6	Change in bowel habits, rectal bleeding	Adenocarcinoma of rectum T4N0M1	No	No	153	94.9	400	
